# Application of Near Infrared Spectroscopy to Monitor the Quality Change of Sour Cherry Stored under Modified Atmosphere Conditions

**DOI:** 10.3390/s23010479

**Published:** 2023-01-02

**Authors:** Gergo Szabo, Flora Vitalis, Zsuzsanna Horvath-Mezofi, Monika Gob, Juan Pablo Aguinaga Bosquez, Zoltan Gillay, Tamás Zsom, Lien Le Phuong Nguyen, Geza Hitka, Zoltan Kovacs, Laszlo Friedrich

**Affiliations:** 1 Department of Postharvest, Commerce, Supply Chain and Sensory Science, Institute of Food Science and Technology, Hungarian University of Agriculture and Life Sciences (MATE), H-1118 Budapest, Hungary; 2 Department of Food Measurement and Process Control, Institute of Food Science and Technology, Hungarian University of Agriculture and Life Sciences (MATE), H-1118 Budapest, Hungary; 3 Department of Livestock Product and Preservation Technology, Institute of Food Science and Technology, Hungarian University of Agriculture and Life Sciences (MATE), H-1118 Budapest, Hungary

**Keywords:** *Prunus cerasus* L., ripeness, MAP, modified atmosphere packaging, NIR spectroscopy, chemometrics

## Abstract

Determining and applying ‘good’ postharvest and quality control practices for otherwise highly sensitive fruits, such as sour cherry, is critical, as they serve as excellent media for a wide variety of microbial contaminants. The objective of this research was to report two series of experiments on the modified atmosphere storage (MAP) of sour cherries (*Prunus cerasus* L. var. Kántorjánosi, Újfehértói fürtös). Firstly, the significant effect of different washing pre-treatments on various quality indices was examined (i.e., headspace gas composition, weight loss, decay rate, color, firmness, soluble solid content, total plate count) in MAP-packed fruits. Subsequently, the applicability of near infrared (NIR) spectroscopy combined with chemometrics was investigated to detect the effect of various storage conditions (packed as control or MAP, stored at 3 or 5 °C) on sour cherries of different perceived ripeness. Significant differences were found for oxygen concentration when two perforations were applied on the packages of ‘Kántorjánosi’ (*p* < 0.01); weight loss when ‘Kánorjánosi’ (*p* < 0.001) and ‘Újfehértói fürtös’ (*p* < 0.01) were packed in MAP; SSC when ‘Újfehértói fürtös’ samples were ozone-treated (*p* < 0.05); and total plate count when ‘Kántorjánosi’ samples were ozone-treated (*p* < 0.01). The difference spectra reflected the high variability in the samples, and the detectable effects of different packaging. Based on the investigations with the soft independent modelling of class analogies (SIMCA), different packaging and storage resulted in significant differences in most of the cases even on the first storage day, which in many cases increased by the end of storage. The soft independent modelling of class analogies proved to be suitable for classification with apparent error rates between 0 and 0.5 during prediction regardless of ripeness. The research findings suggest the further correlation of NIR spectroscopic and reference parameters to support postharvest handling and fast quality control.

## 1. Introduction 

*Prunus avium* L. and *P. cerasus* L. (sweet and sour cherries) from the *Prunus* genus are among the earliest domesticated and most widely commercialized fruit crops. The varieties belonging to these are of culinary and industrial importance and can be found on market shelves in both fresh and processed form, according to their intended use [1]. The consumer perception of sour cherries acknowledges that the stalk, size, shape, color, texture, overall flavor and sweetness-sourness are particularly important attributes [2]. The sugar-organic acid ratio in the fruits plays a key role in flavor development, indirectly contributing to consumer loyalty and trust and in the product. In addition, sour cherries are a rich source of bioactive secondary metabolites and phenolic compounds also known as natural antioxidants [3], which places them in the category “super food” [4]. The preferential composition and soft tissue structure make the fruits particularly sensitive to external impacts, so they perish easily during their otherwise short shelf life (7–14 days) [5]. 

During its development and maturation, the fruit ripens and reaches the stage where it is ready for consumption. In contrast with the quantitative increase in growth, ripening is a qualitative change, during which biochemical transformations take place in the fruit. In the post-harvest period, stone fruits have a minimal post-ripening ability, which significantly affects their post-harvest handling, cold storage and shelf life [6]. One of the most common physiological behaviors allowing the comparison and characterization of fruits is respiration and the chemical, biochemical and physiological changes associated with it [7]. The respiratory intensity of non-climacteric respiratory fruits is constantly decreasing during their development, and therefore they are unable to ripen more [8]. 

To reduce fruit production losses, several research studies have focused on the combined development and application of proper varieties and post-harvest technologies, namely determining the optimum harvesting time, refrigeration and controlled and modified atmosphere storage. Seemingly, the level of fruit maturity is the basis of the ‘chain’, since by determining it one can infer the time of harvest and expected shelf life. Empirical and destructive methods and indices for determining ripeness are widely used by fruit producers and processors. Such quality indicators include detachment and tissue firmness [9], color [10], dry matter (DM), water-soluble solids (SSC), titratable acid (TA) and phenolic content [11]. 

Among the available post-harvest technologies, several new washing technologies have been introduced in the food industry. Micro- and nano-bubbles are used in the engineering, agriculture, environmental protection, food and pharmaceutical industries. Microbubbles are characterized by their small size, one thousandth the size of a mm in diameter, large surface area, high biological activity, low frictional resistance, high internal pressure, high gas dissolution capacity and high curvature stress [12]. The smaller the diameter of the bubble, the higher the specific surface to volume ratio and the higher the density of bubbles per constant volumetric flow rate [13]. Ozone, triatomic oxygen (O_3_), is a highly reactive compound with potential micro-bubbling activity. Ozone effectively eliminates a broad spectrum of microorganisms by strongly oxidizing their cell membranes and in addition leaves no by-products after decomposition that are harmful to living organisms. The results of using ozone to inhibit microbial growth on strawberries demonstrated the superior efficacy of microbubble ozone washing [14]. 

Modified atmospheres (MA) in the form of elevated concentrations of carbon dioxide (CO_2_), and reduced levels of oxygen (O_2_) and ethylene (C_2_H_4_), can be advantageous supplements to provide optimum temperature and relative humidity (RH) in maintaining the quality of fresh fruits and vegetables after harvest [15]. The optimal cold storage conditions for sour cherry are 0–1 °C and 90–95% relative humidity. In a controlled atmosphere, 0–1 °C, 1–2% O_2_ and 20–25% CO_2_ may be preferable in some cases for spoilage inhibition compared to the 3–4% O_2_ and 2–3% CO_2_ found in the literature. Shelf-life can be increased up to 1–3 weeks in a normal atmosphere, and 4–6 weeks in controlled atmosphere conditions. In a related study, harvested fruits of Hungarian sour cherry cultivars (‘Érdi jubileum’ and ‘Érdi bőtermő’) were stored at 0 °C in MAP (15% O_2_, 10% CO_2_ and 75% N_2_) [16]. In another study, the effects of three gas compositions (ambient, 5% O_2_ + 5% CO_2_ + 90% N_2_ and 10% O_2_ + 15% CO_2_ + 75% N_2_) and two storage temperatures (0 and 5 °C) were compared. The results indicated a better preservation of qualitative properties such as weight loss, tissue firmness and color in a modified atmosphere; in 5% O_2_ + 5% CO_2_ + 90% N_2_ the lowest weight loss and the highest firmness were obtained [17]. According to another study, the death rate of *Salmonella enteritidis* on the surface of tomatoes stored under MAP conditions (6% O_2_, 4% CO_2_) was faster than that of *S. enteritidis* stored in air [18]. 

Due to the complex physical and chemical nature of sour cherries, there is as yet no universal marker that can cover the physiological state of the fruits; therefore, correlative fingerprinting techniques are used, which can give a complete analytical print of the fruit with a single measurement. With almost a hundred years of history in agricultural research, fast and non-destructive near-infrared (NIR) spectroscopy combined with chemometric methods is widely used to monitor and predict fruit maturity, quality attributes and storage based on spectral patterns [19]. Some of the most recent studies specifically conducted on cherries are the following. 

Vitalis et al. used the NIR technique in the wavelength range of 950–1650 nm to classify sweet and sour cherry varieties according to perceived ripeness. The classification accuracies were observed to be dependent on whether the spectra were recorded on the ripe or unripe sides of the fruits. Linear discriminant analysis (LDA)-based ripeness models resulted in more than 78% correct classification during model validation [20]. Li et al. applied hyperspectral imaging (I) capable of the spatial-spectral mapping (874–1734 nm) of the fruit surface to classify cherries according to ripeness stage and estimate SSC and pH distribution among samples, where 96.4% classification accuracy was achieved with LDA. Ratios of the standard deviation of the prediction set to the standard deviation of the prediction error (RPD) of 2.7 for SSC and 2.4 for pH were obtained, when the combination of genetic algorithm (GA) variable selection and multiple linear regression (MLR) was employed on the NIR spectra [21]. Results of NIR spectroscopic measurements (729–975 nm) performed at 0 and 23 °C demonstrated robustness, when DM and SSC were evaluated, and a good correlation with sensorial traits [22]. Fodor developed classification models based on the reference parameters of sweet and sour cherry varieties, including DM, SS, TA, anthocyanin content and SSC/TA; the latter distinguished mature and immature categories with an accuracy of 98.44% [23]. 

Several studies have investigated the effect of MAP on cherry variety storability and shelf life extension, mostly using reference methods [24,25,26,27]. Limited NIR spectroscopic results are available on the modified storage of cherries, though not so for stone fruits and controlled storage. In connection with plums, Guo et al. effectively applied NIR spectroscopy combined with qualitative and quantitative chemometric modelling for the fast determination of optimal storage time [28]. Vitalis et al. have repeatedly applied NIR spectral monitoring for controlled refrigerated and ambient storage, besides the detection of brown rot caused by *Monilinia* on plums [29,30] and sour cherries [31]. 

However, only a few examples can be found on the state-of-the-art spectral characterization of the modified atmosphere storage of stone fruits, especially sour cherries, to the best of our knowledge. This study, therefore, aimed to investigate the effects of different pre-treatments on quantitative and qualitative properties with reference measurements, as well as to discover detectable changes with the fingerprinting approach of NIR spectroscopy during the MAP storage of sour cherries. 

## 2. Materials and Methods 

To meet the objectives, the research was divided into two sections. In Experiment I, the effects of different pre-treatments and modified atmosphere packaging on the reference properties of sour cherries were monitored. In the following Experiment II, using the treatment proved to be optimal, the detectable spectral changes during MAP storage were investigated on fruits of different perceived ripeness. 

### 2.1. Experiment I 

#### 2.1.1. Sour Cherry Samples and Their Treatment 

##### Materials

‘Újfehértói fürtös’ (UF) and ‘Kánorjánosi’ (KJ) sour cherries (*Prunus cerasus* L.) involved in the research were harvested at commercial ripeness on an orchard in Csegöld (Szabolcs-Szatmár-Bereg county, Hungary). 

##### Sample Preparation 

Prior to sample preparation, all working surfaces, chopping boards, containers and other tools were washed and sanitized with 200 μL/L sodium hypochlorite solution. 

The sour cherries were pre-selected according to uniform weight (5–8 g/fruit) and tangible firmness. Fruits without visible physical damage and fungal infections were stored at 2 °C and 90% RH before processing. The total sample quantity was divided into two sub-groups, for which two types of washing technology (with tap water, marked as W; with ozone (O_3_) microbubble liquid, marked as O) were applied. The samples were washed in 70 L of washing liquid and fresh water was used between washes. 

The ozone generator used for the production of ozone microbubble liquid (GO-R 5G, Guangzhou Ozone Environmental Technology Co., Ltd., Guangzhou, China) produced ozone with a volumetric flow capacity of 100 L/h and a concentration of 140–190 ppm. The amount of ozone produced per hour, given in g by the ozone generator, can be converted to ppm using the following correlations (Equation (1)):

1 g O_3_/m^3^ = 467 ppm O_3_
0.4 g O_3_/m^3^ = 467 ×0.4 = 187~190 ppm O_3_
100 L h^−1^ × 190 ppm = 19 mg O_3_/h (1)

The generated ozone concentration was determined using an ozone measuring device (Ozone Analyzer BMT-963, BMT MESSTECHNIK GmbH, Stahnsdorf, Germany). The microbubble generator (Gas-liquid mixing pump, Type: YL8022, Model: 25GO-2SS, Guangzhou Ozone Environmental Technology Co., Ltd., China) was used for producing bubbles with a diameter of 20–30 µm according to the company. 

**Figure 1:**
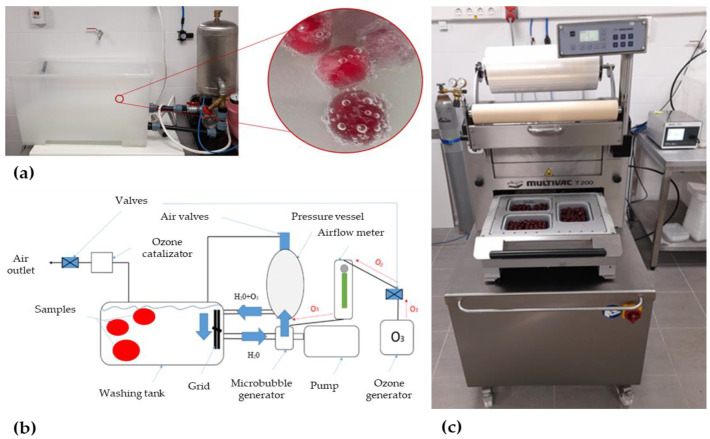
Washing of sour cherry with ozone microbubbles (**a**); Technical diagram of ozone washing (**b**); Sour cherry modified atmosphere packaging in progress (**c**).

The ozone generator has a built-in pump. The ozone passes through an airflow meter (airflow: 100–120 L/h) and directly connects to the mixing point. The pump of the microbubble generator sucks the tap water through a pipe from the wash tub; thus, the liquid and gas are mixed in the rotating generator. Through the pressurized mixing tank, the microbubble water is returned to the wash tub, where the washing treatment starts. After a few minutes, the microbubbles become visible and the water becomes milky opalescent. Figure 1a,b present the process of ozone washing. 

##### Sample Packaging

After washing, sour cherries were placed into a polypropylene tray (Linpac Packaging Kft., Törökbálint, Hungary) of 18.7 cm length × 13.7 cm width × 3.6 cm depth. The trays were covered with foil (Opalen layer thickness: 65 µm, type: HB AF PP width: 420 mm, Multivac, Germany) (Figure 1c). 

The initial gas concentration was predefined and used in a modified atmosphere (5% O_2,_ 15% CO_2_, 80% N_2_). The trays were flushed for 10 min with the set gas combination, which was mixed with a Dansensor unit (MAP Mix Provectus 3-gas mixer, MOCON Europe A/S, Ringsted, Denmark) for which Linde Gas Hungary (Répcelak, Hungary) supplied the CO_2_ gas (BIOGON C), and nitrogen gas was produced (nitrogen purity: 99.5%) by a nitrogen generator, model: UHPLCMS 12E (Domnick Hunter Gas Generation Division, Gateshead, UK). Gas compounds generally were unable to diffuse in and out of this packaging material. Samples were continuously processed to avoid excessive exposure of the fruit to air. 

The packages prepared were randomly split into two sub-groups. A different number of perforations (1 or 2) were applied for the sub-groups. The lid film was perforated with one or two micro-perforations of 200 µm in diameter using a needle. Control samples were only placed on a tray without washing and packaging. The preparation resulted in a total of 5 sample groups per variety (Table 1), where each tray contained 150 g ± 2 g of sour cherries. All samples were stored at 2 ± 0.5 °C in the dark for fourteen days. 

#### 2.1.2. Reference Measurements 

Trays containing the samples pretreated in different ways were randomly taken on day 0 and then the 2nd, 5th, 7th, 9th, 12th and 14th days of storage for analyses. The methods used included non-destructive and destructive, physical, chemical and microbiological tests. Gas concentrations in the pack, weight loss, decay rate, texture analysis (flexible firmness by Durofel, SETOP Giraud Technologie, Cavaillon, France), color, water-soluble solid content and total plate count were determined. Three packs were used as measurement replicates.

##### Determination of Headspace Gas Concentration

Three trays per group were randomly withdrawn from storage and the headspace gas concentrations of O_2_ and CO_2_ in the package were measured. A needle was pierced into the package, and then gases were pumped out through the needle and injected into the gas analyzer (WITT Oxybaby 6i O_2_/CO_2_, WITT-Gasetechnik, Witten, Germany). Results were expressed as percentages of O_2_ and CO_2_ inside the package [32]. To calculate the time dependence of gas concentration change, the Gompertz model was applied, where the following factors were defined (Equation (2)).

(2) $$f\left(t\right)=ae^{-e^{b-ct}}$$

where: 

*a*—is an asymptote;

*b*—sets the displacement along the x-axis (translates the graph to the left or right);

*a*—sets the growth rate (y scaling);

*e*—is Euler’s Number (*e* = 2.71828…).

##### Determination of Weight Loss 

Weight loss was determined by weighing the packages at the beginning and at each measurement date with an electronic balance (BP 210 S, Sartorius AG, Göttingen, Germany). Results were expressed as percentage loss of initial readings [32] (Equation (3)).

(3) $$\mathrm{Weight}\;\mathrm{loss}\;\left[\%\right]=\frac{W_{day_{n}}}{W_{day_{0}}}\times 100\;$$

where: 

$W_{day_{0}}\;$ is the initial net weight of a certain package; 

$W_{day_{n}}\;$ is net weight of a certain package analyzed on a certain day of measurement. 

##### Determination of Decay Rate

After the determination of weight loss, the decayed pieces were selected visually. The weights of the acceptable sour cherry samples were measured with an electronic balance (BP 210 S, Sartorius AG, Göttingen, Germany). Results were expressed as the decay percentages of the initially measured weight (Equation (4)).

(4) $$\mathrm{Decay}\;\mathrm{rate}\;\left[\%\right]=\frac{M_{day_{n}}}{W_{day_{0}}}\times 100\;$$

where: 

$W_{day_{0}}\;$ is the initial net weight of a certain package; 

$M_{day_{n}}\;$ is the net weight of acceptable fruits in a certain package analyzed on a certain day. 

##### Determination of Color 

The color of the sour cherry was measured with a Minolta Chroma Meter CR-400 (Minolta Corporation, Osaka, Japan). Standard CIE L*, a* and b* color parameters were determined at one point on each piece on the darkest surface.

##### Determination of Firmness 

The firmness of the sour cherries was measured with a Durofel quality control device (SETOP Giraud Technologie, Cavaillon, France). The firmness of samples was determined using a probe of 5 mm in diameter. The maximum force used to penetrate the fruit was recorded in the Durofel Index (0–100 value). One point of each sample was measured randomly. Twenty samples were used as replicates.

##### Determination of Total Soluble Solid Content 

Total soluble solid content (TSS; Brix°) was obtained with a digital refractometer (ATAGO Pocket PAL-1, ATAGO CO. Ltd., Tokyo, Japan) using the squeezed juice of sour cherry [32].

##### Determination of Total Plate Count 

Microbiological sampling was performed in three series. A total of 3–3 sour cherries per the washing treatment group were taken immediately after washing (initial), and then 3–3 samples were taken after 1 and 2 weeks of storage. The colony counting procedure was performed in the following steps:

- A metal ring with an inner diameter of 1 cm was sprayed with a 70 v/v% alcohol solution using tweezers and then sterilized over a flame.
- The ring was placed on the surface of the sour cherry so that the sampling area was a constant, and a sterile piece of gauze (Mulltupfer EN 14079 Typ 20, NOBA Verbandmittel Danz GmbH, Wetter (Ruhr), Germany) was pressed onto the area inside the ring using sterilized forceps.
- The samples (pieces of gauze) were soaked in 9 mL of peptone water for 5 min, shaking the solution at intervals to transfer bacteria and fungi into the solution as efficiently as possible.
- After preparing a decimating dilution series from the resulting sample solution, plate casting was performed with Nutrient Agar (Biokar Diagnostics, Allonne, France).
- After the solidification of the agar, the Petri dishes were incubated at 30 °C for 24 h in a thermostat and the total aerobic mesophilic colony count was determined from the dishes with a colony count between 30 and 300 using a colony counter (BZG 30, WTW, Weilheim in Oberbayern, Germany).

#### 2.1.3. Univariate Data Analysis 

Results of the reference measurements were processed with Microsoft Excel 2011 (16.0.15831.20098) and IBM SPSS v27 statistical software and were then evaluated with univariate data analysis. First, descriptive statistics, mean and standard deviation (SD), were used for the primary characterization and illustration of changes in quality traits of the stored samples. Then, a two-way analysis of variance (ANOVA) was employed to determine whether washing treatment, packaging and their interaction (treatment × packaging) had significant effect on the investigated reference parameters. In case of significant ANOVA (*p* < 0.05), pairwise comparisons of factor levels (treatments and packaging) were performed using Tukey’s test when the assumption of homogeneity of variance was met, and the Games-Howell’s test was used when homogeneity of variance did not meet. 

### 2.2. Experiment II

It is known from the literature that good results can be achieved with NIR spectroscopy-based modelling when the prediction of internal quality of fruits is in focus; however, the fingerprinting approach of the NIR technique was applied with the purpose of discriminating between fruit ripeness and different treatments without direct knowledge of these parameters. The knowledge gained in Experiment I formed the basis for Experiment II, when that combination of washing procedure and package perforation number was utilized that was closest to industrial practice, and was found to be optimal; i.e., the final physiological state of the samples was almost equal to the initial, based on the reference results. 

#### 2.2.1. Fruits and Their Pretreatment 

The above-mentioned sour cherry varieties, ’Kántorjánosi’ (KJ) and ‘Újfehértói fürtös’ (UF), were involved in a second experimental setup as well. The fruits were harvested in Csegöld (Szabolcs-Szatmár-Bereg county, Hungary). A total of 300 fruits per variety were manually divided equally into three categories according to their perceived ripeness. The following ripeness levels were determined:

- L1: fruits considered as less ripe;
- L2: fruits considered fairly ripe;
- L3: fruits considered as ripe.

After sorting, the fruits were detached from the stalk and were washed with tap water as this washing treatment proved to be the most applicable based on preliminary results. Subsequently, half of the fruit volume (150 samples per variety) was subjected to modified atmosphere packaging, thus forming the MAP sample group. The process of packaging and the gas composition were the same as described in Section 2.1.1. Two perforations were applied to the packaging foil for each package since, based on preliminary results, this proved to be the best. The samples belonging to the other half were packed without further treatment, forming the control sample group. Half of the packed fruits were then exposed to controlled storage at around ~3 °C or ~5 °C for 10 days. Identically prepared but different samples were analyzed through the storage because at the end of each measurement samples were saved for later use. To ensure a sufficient sample quantity for the non-destructive measurements, five parallel sour cherries of the same variety and perceived ripeness were packed at once in each sample group for each measurement day. Table 2 summarizes the resulting groups of samples. 

#### 2.2.2. Acquisition of the Spectra 

For the non-destructive examination of sour cherries packed and stored under different environmental conditions, samples were subjected to near-infrared scanning applying XDS RapidContent Analyzer (Metrohm, Herisau, Switzerland) benchtop spectrometer with 0.5 nm resolution. The spectral data were collected in the wavelength range of 400–2500 nm by scanning the five parallel samples of each sample group placed in a glass sample holder (45 mm in diameter, 40 mm in height) at the same time, and three successive scans were performed. 

The device recorded the spectra of the samples in a reflectance arrangement. During the measurements, illuminating light coming from the monochromator was transmitted through the bottom of the vessel filled with fruit, placed and positioned with a sample centering iris in the instrument, which was reflected, scattered, transmitted or absorbed, depending on the material characteristics of the fruits to be analyzed. The light coming back from the vessel was detected by a detector installed in front of the monochromator, which yielded the absorbance at each wavelength, i.e., the sample spectrum. The acquisition of the spectra was performed on the 1st, 3rd, 6th, 8th and 10th days of refrigeration. At the end of storage, a total of 360 spectra (2 × 12 sample groups × 3 scanning × 5 measurement days) were further evaluated. 

#### 2.2.3. Data Analysis 

The evaluation of the NIR spectra was performed using R-project v 3.6.3 and aquap2 package [33]. The data were evaluated separately by sour cherry variety. 

The NIR spectra were analyzed in the wavelength range of 1100–1850 nm. After the elimination of outliers to reduce unwanted effects experienced during spectrum acquisition and occurring in the NIR spectra, pretreatments were applied, namely, Savitzky-Golay smoothing filter (2nd order polynomial, 33 data point frame) [34] followed by multiplicative scatter correction (MSC). Multiplicative scatter correction proved to be the most effective of the many spectral pretreatments used for the elimination of observed baseline shifts. Before spectrum pretreatment, the NIR data were filtered according to the sample group to be analyzed in all cases. 

The NIR spectral data analysis commenced with the visual inspection of the raw and difference spectra, which were calculated by subtracting the pretreated and averaged spectra collected on the 1st storage day from the identically pretreated and averaged spectra collected on all the storage days (Equation (5)). To discover wavelengths covering notable absorbance differences among varieties caused by the different storage conditions, the major peaks of the difference spectra were investigated.

(5) $$\mathrm{diff}.\;S=\bar{S_{{\mathrm{day}}_{n}}}-\bar{S_{{\mathrm{day}}_{1}}}$$

where: 

$\bar{S_{{\mathrm{day}}_{n}}}$ is pretreated and averaged spectrum of a certain sample group recorded on a certain measurement day; 

$\bar{S_{{\mathrm{day}}_{1}}}$ is pretreated and averaged spectrum of a certain sample group recorded on day 1; diff. S is the calculated difference spectra. 

After the preliminary spectral inspection, supervised classification modelling was used to determine how the difference between each group of samples changes with storage time, and also to assess whether there was a detectable difference between the ripeness levels, packaging and storage conditions on the initial and final days of storage. For this purpose, as well as to achieve robust classification, soft independent modeling of class analogies (SIMCA) was applied [35], which is widely used in NIR spectroscopy-based food research due to its proven efficiency [36,37]. This chemometric method models the multivariate space occupied by a class and determines whether an observation belongs to it or not based on calculated interclass distances and variable importance. The latter gives the discriminating power of variables (i.e., wavelengths) contributing significantly to the discrimination of classes and the identification of descriptive absorption bands relating to spectral difference and changes in sour cherries. The modelling was performed on the smoothed and MSC-treated spectral data filtered for different sample groups, where the class variables were the factor levels (i.e., storage days, ripeness levels, mode of packaging, storage temperature). The number of components that enabled the best possible discrimination was denoted by k in the manuscript. In the analysis, 50% of the data were used during the calibration, and the remaining 50% were projected into the constructed SIMCA model during validation. 

## 3. Results and Discussion 

### 3.1. Results of Experiment I

This section summarizes the differences that were detected during the 14 days of the modified atmosphere storage of sour cherries washed and packed in different ways, based on the results of the reference measurements. 

#### 3.1.1. Results of Headspace Gas Concertation 

**Figure 2:**
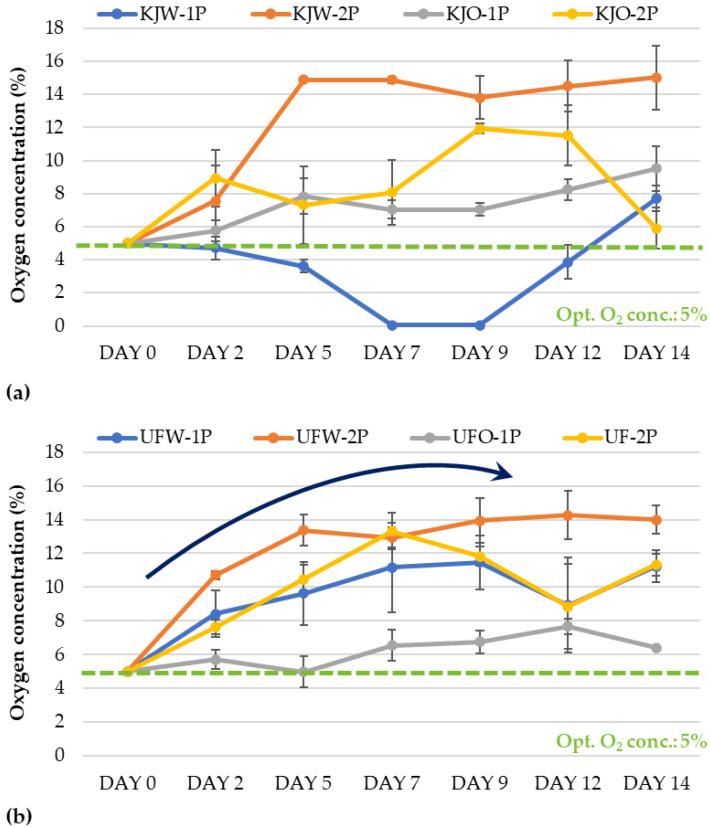
Change in headspace oxygen concentration in modified atmosphere packages of (**a**) ‘Kántorjánosi’and (**b**) ‘Újfehértói fürtös’ sour cherries with different washing pretreatments.

The change in oxygen concentration was monitored for samples stored under MAP conditions. In the case of the ‘Kántorjánosi’ variety (Figure 2a), the oxygen concentration did not exceed 10% for packages with one perforation at any time during the 14 days of storage. However, a 15% oxygen concentration was detected when two perforations were used. No significant differences were observed between washing treatments, while the application of perforations had a significant effect (*p* < 0.05) on the change in oxygen concentration during storage. Based on these results, we would recommend washing with ozone microbubbles using one perforation to control the O_2_ headspace concentration during the storage of the ‘Kántorjánosi’ sour cherries, since this handling was able to retain the initial oxygen concentration best. 

In the ‘Újfehértói fürtös’ samples, a saturation trend was observed in oxygen concentration (Figure 2b). There was no significant correlation between the samples washed with tap water or with ozone microbubbles. In this case, the number of perforations also induced variation. The samples washed with ozone microbubbles and packed with one perforation could maintain their initial oxygen gas concentration throughout the storage period. The initial average changed from 5% to 6.42% at the end of day 14. 

**Figure 3:**
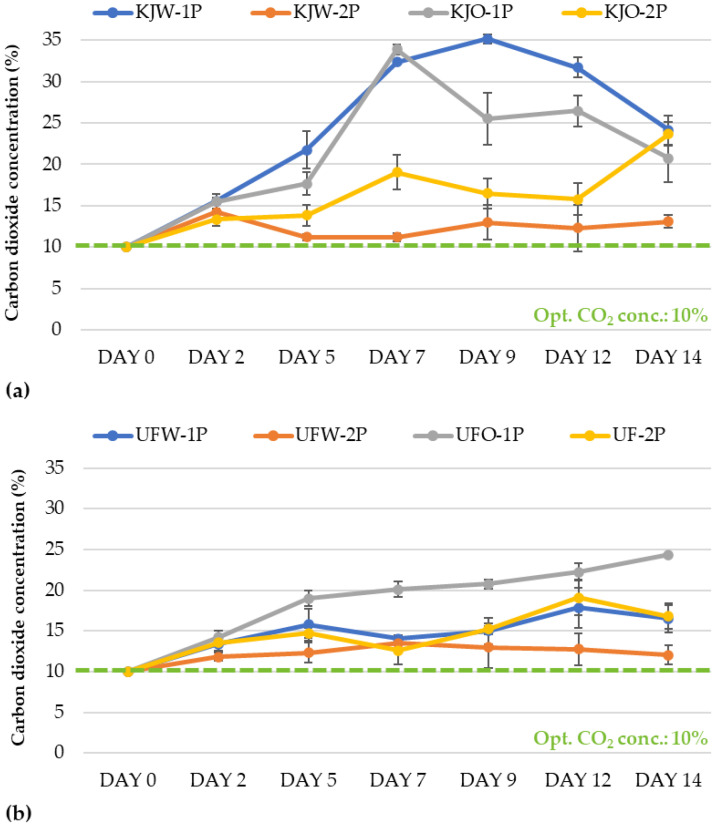
Change in headspace carbon dioxide concentration in modified atmosphere packages of (**a**) ‘Kántorjánosi’ and (**b**) ‘Újfehértói fürtös’ sour cherries with different washing pretreatments.

Due to its important role in respiratory metabolism, the change in carbon dioxide concentration was also investigated in the case of the modified atmosphere packages (Figure 3). The initial 10% CO_2_ concentration was maintained with two perforations for the ‘Kántorjánosi’ samples washed with tap water (KJW-2P). The CO_2_ concentration increased slightly from 10% to 13.1% by the end of storage. The application of two perforations achieved better results for the ozone microbubble-treated fruit as well. In the case of a single perforation, the CO_2_ concentration reached values of 30–35%, which already has harmful effects. A high concentration of CO_2_ induces diffusion into the tissue and acidifies it, which can lead to unpleasant sensory properties. It can be concluded that in the case of ‘Kántorjánosi’ sour cherries, two perforations should be applied when modified atmosphere packaging is applied. The washing method did not result in a significant difference (Figure 3a). 

The evaluation of the CO_2_ concentration in the headspace of the ‘Újfehértói fürtös’ samples also showed better results when two perforations were used during packaging. Interestingly, samples with one or two perforations after washing in tap water did not show much difference (UFW-1P vs. UFW-2P) (Figure 3b). 

In the comparison of the two sour cherry varieties, the results showed that the ‘Kántorjánosi’ variety had a higher respiration intensity, so two perforations are recommended for this variety to maintain the initial gas concentration in the modified atmosphere packages. 

#### 3.1.2. Results of Weight Loss 

**Figure 4:**
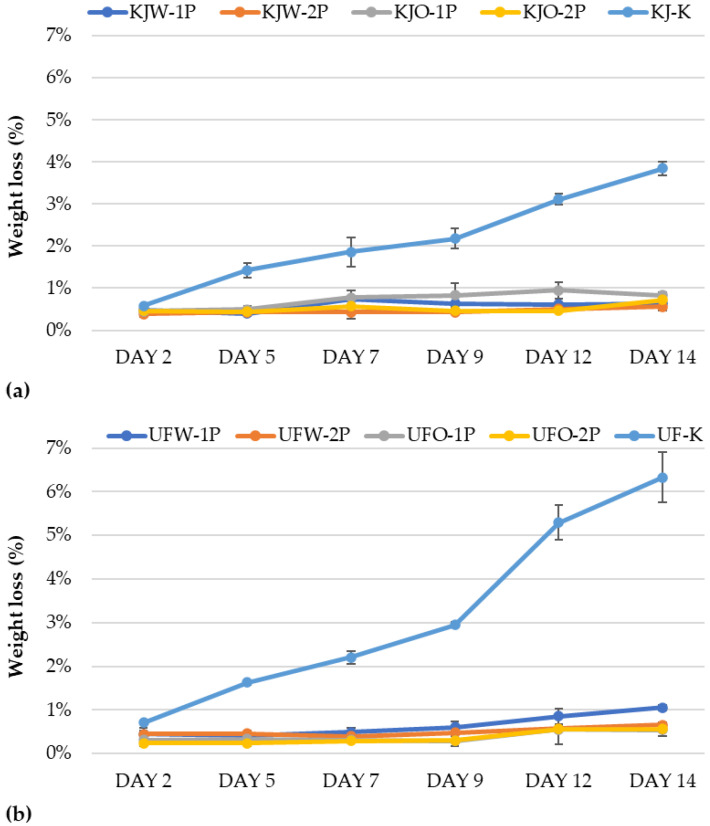
Weight loss during the storage of control and MAP (**a**) ‘Kántorjánosi’ and (**b**) ‘Újfehértói fürtös’ sour cherries with different washing pretreatments.

The weight loss gave predictable results, as the control samples of ‘Kántorjánosi’ (Figure 4a) and ‘Újfehértói fürtös’ (Figure 4b) sour cherries, reached 3.9% (KJ-K) and 6.3% (UF-K) by the end of storage, respectively. In contrast, the treated and packaged products (KJW-1P; KJW-2P; KJO-1P; KJO-2P; UFW-1P; UFW-2P; UFO-1P; UFO-2P) showed approximately 1% weight loss during storage. No significant differences were observed within the washing treatment and packaging groups. Based on the results, it is recommended to pack fruits in a modified atmosphere to prevent significant weight loss during storage. 

#### 3.1.3. Results of the Decay Rate 

The analysis of the deterioration rates included in the Supplementary Materials revealed a similar trend to the weight loss results. While the control samples of the ‘Kántorjánosi’ sour cherry (KJ-K) increased beyond 30% by the end of storage, the spoilage rate of the treated and packed (KJW-1P; KJW-2P; KJO-1P; KJO-2P) samples reached a 7% maximum (Figure S1a). The trend in the percentage of deterioration of the ‘Újfehértói fürtös’ cherries was similar to that of the ‘Kántorjánosi’ samples. Here, the percentage of spoilage of the control sample (UF-K) was 56% at the end of the experiment, while the maximum value of the washed and packed samples (UFW-1P; UFW-2P; UFO-1P; UFO-2P) was 12.9%. It is suggested to pack fruit to significantly decrease the decay rate (Figure S1b). 

#### 3.1.4. Results of Color Measurements 

During the color measurements, CIE standard L*, a*, b* and h values were recorded on the fruit surface. The assumptions for performing ANOVA were met only for the ‘Újfehértói fürtös’ samples, and there only for the L* and b* values; therefore, these results could be further evaluated and discussed (see Supplementary Materials). Significant differences were observed for the L* values on days 5 and day 9 for UFW-2P compared to the other samples, indicating that these samples were generally darker (Figure S2). 

The b* color value represents the blue-yellow color shift, where negative values indicate bluish coloring and positive values indicate a yellowish color. Overall, a slight decrease in b* coloration was observed during storage, which may be attributed to the accumulation of bluish compounds in the plant tissues. It was found that the samples washed in tap water (UFW-1P; UFW-2P) were dominated by the yellow component, while the other treatments (UFO-1P; UFO-2P; UF-K) were characterized by the blue component (Figure S3). 

#### 3.1.5. Results of Firmness 

**Figure 5:**
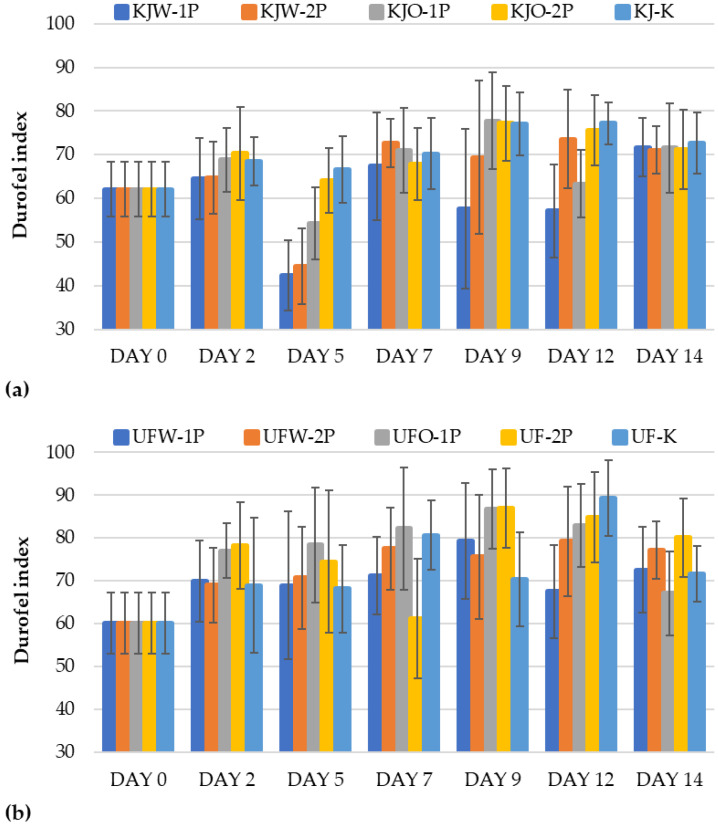
Change in firmness (Durofel Index) during the storage of control and MAP: (**a**) ‘Kántorjánosi’ and (**b**) ‘Újfehértói fürtös’ sour cherries with different washing pretreatments.

The firmness was analyzed by measuring the change in elastic hardness of the fruits during the 14 days of storage. In the case of the ‘Kántorjánosi’ variety (Figure 5a), the fruit hardness increased for the control samples (KJ-K). This can be attributed to the fact that as the moisture content of the fruits decreased, their firmness may have increased in proportion. However, no significant differences were observed; therefore, no comparisons could be made on the effectiveness of the washing treatments and packaging (i.e., the number of perforations). Similar results to the former could be reported for the ‘Újfehértói fürtös’ variety (Figure 5b). No definite conclusions can be drawn regarding the use of different washing treatments or package perforations.

#### 3.1.6. Results of Soluble Solid Content 

**Figure 6:**
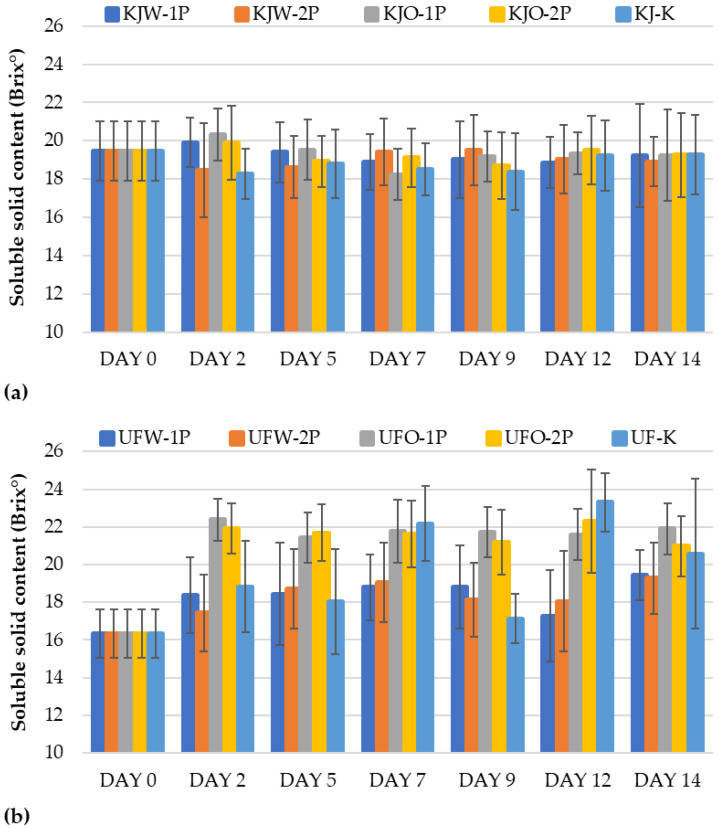
Change in soluble solid content (Brix˘) during the storage of control and MAP: (**a**) ‘Kántorjánosi’ and (**b**) ‘Újfehértói fürtös’ sour cherries with different washing pretreatments.

Another critical feature for the sensory evaluation of sour cherries is the soluble solid content, which allows us to infer the sweetness of the fruit. Interestingly, no statistically detectable change was observed in SSC during storage for the ‘Kántorjánosi’ variety. Regardless of the washing and packaging method, the composition remained almost constant (Figure 6a). Davarynejad and Aryanpooja [16] had similar findings when they compared modified and ambient atmosphere storage. The SSC of the sour cherries did not change much (ambient air: 22.5 Brix°; modified atmosphere: 21.3 Brix°); however, their results showed a significant difference. 

In the case of the ‘Újfehértói fürtös’ samples, the SSC showed an increasing trend during storage. The samples washed in tap water (UFW-1P; UFW-2P) were generally characterized by a smaller increase in SSC compared to the ozone-treated ones (UFO-1P; UFO-2P) that reached up to 4 Brix°, higher than the control ones. A significant difference was found for this sample set (Figure 6b). 

#### 3.1.7. Results of Total Plate Count 

**Figure 7:**
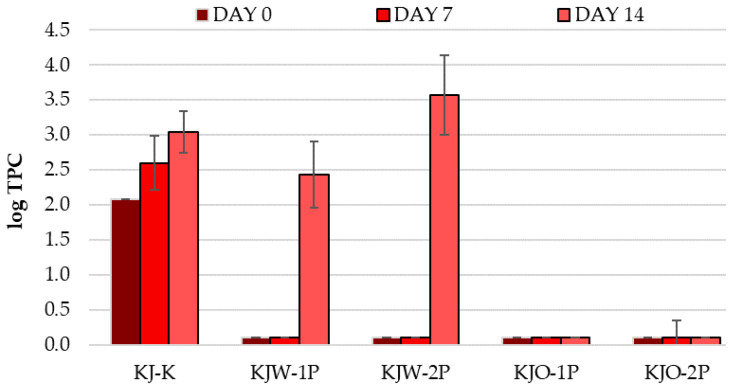
Change in total plate count (TPC) during the storage of control and MAP ‘Kántorjánosi’ sour cherries with different washing pretreatments.

The degree of the microbiological contamination of the fruit surface is crucial. When determining the expected shelf life of the sour cherry, the total plate count (TPC) provides a good basis for this. Interpretable results in TPC were obtained only in the case of the ‘Kántorjánosi’ variety, as illustrated in Figure 7. The TPC of the control sample (KJ-K) gradually increased from log 2.08 to log 3.04 during the 14 days of storage. This represented a 0.96-fold increase in microbial count. For samples washed in tap water (KJW-1P, KJW-2P), day 0 and day 7 TPC results indicate that this treatment may be an effective method if the fruits are further processed within a short time, since the surface microbial count had grown sharply by day 14 up to log 2.43 and log 3.58 for one- and two-package perforations, respectively, even exceeding the control samples (KJ-K). Ozone washing has been proven to be the most effective in ensuring a minimum microbial count, as it destroys the surface microbes. It can be concluded that the quality of the washing treatment had a strong influence on the control of the surface TPC in the samples tested. 

### 3.2. Results of Experiment II

Based on the findings of the first experiment, tap water washing and two perforations were found to be the most suitable treatments. During the preparation for the second experiment, sour cherries of different varieties and perceived ripeness were packaged accordingly. The samples were stored under different refrigerated conditions at around 3 or 5 °C. A benchtop NIR spectrometer was employed to monitor changes based on spectral patterns during 10 days of storage, which allowed non-destructive, non-invasive investigations. 

#### 3.2.1. Inspection of the Difference Spectra 

The principal aim of controlled refrigerated storage is to preserve the initial physiological state of the fruits for as long as possible, also supported by results obtained from the reference methods, which also imply minimal differences between the spectra. 

**Figure 8:**
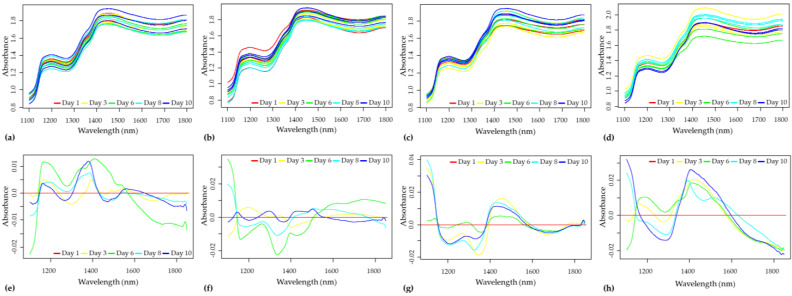
Raw spectra collected on ‘Kántorjánosi’ samples during the 10 days of storage: (**a**) Control samples stored at 3 °C; (**b**) Control samples stored at 5 °C; (**c**) MAP samples stored at 3 °C; (**d**) MAP samples stored at 5 °C. Average difference spectra of ‘Kántorjánosi’ samples calculated on storage days: (**e**) Control samples stored at 3 °C; (**f**) Control samples stored at 5 °C; (**g**) MAP samples stored at 3 °C; (**h**) MAP samples stored at 5 °C.

The raw spectra collected over 10 days from sour cherries stored under different conditions are presented in Figure 8a–d for the ‘Kántorjánosi’ variety and Figure S4a–d for the ‘Újfehértói fürtös’ variety, respectively. Prior to spectrum averaging and subsequent subtraction, the spectral data were smoothed and corrected with MSC, as it was found to be the most appropriate spectral treatment for the elimination of observed baseline differences among several pretreatments tested. The differences between spectra recorded on the first and all the measurement days are presented in Figure 8e–h for the ‘Kántorjánosi’ variety and Figure S4e–h for the ‘Újfehértói fürtös’ variety, respectively. For all sample groups, the spectra were averaged, and their difference was calculated by the average spectrum of day 1 from the average spectra of the measurement days. Overall, few trends emerge from studying the difference spectra, but it is clear that the wavelength range of 1350–1450 nm shows markedly significant differences between storage days. Bands at this region are attributed to the absorption of weakly bound water (e.g., water vapor, proton hydrates, water hydration shell, etc.) indicating the respiratory activity of the fruits packed [38]. 

In the case of the ‘Kántorjánosi’ variety, the shape and the peaks of the difference curves clearly show which samples were packed in MAP (Figure 8). Around 1450 nm, MAP-packed samples show positive peaking by the end of storage, while control samples present negative or close to zero values. These similarities were also observed in the ‘Újfehértói fürtös’ variety; however, by day 10, the MAP samples stored at 5 °C were very similar to the initial results (see Figure S4). These findings suggest the focused and supervised analysis of the sub-selected datasets. 

#### 3.2.2. Analysis of Differences between Sample Groups with SIMCA 

After the preliminary inspection of the spectral data, in the interests of robustness, commonly applied SIMCA was used because of the relatively small number of spectra. This supervised chemometric method calculated the differences between the class variables of different sample groups, and the most influential variables (i.e., the absorption bands) at which the variance contributed most to the discrimination. 

##### Discrimination according to Storage Time 

Firstly, SIMCA was performed to classify observations (spectra) according to the day of measurement. In this case, data were filtered to a specific variety, packaging conditions, to investigate the extent to which storage had a detectable effect on the sour cherry samples. 

**Figure 9:**
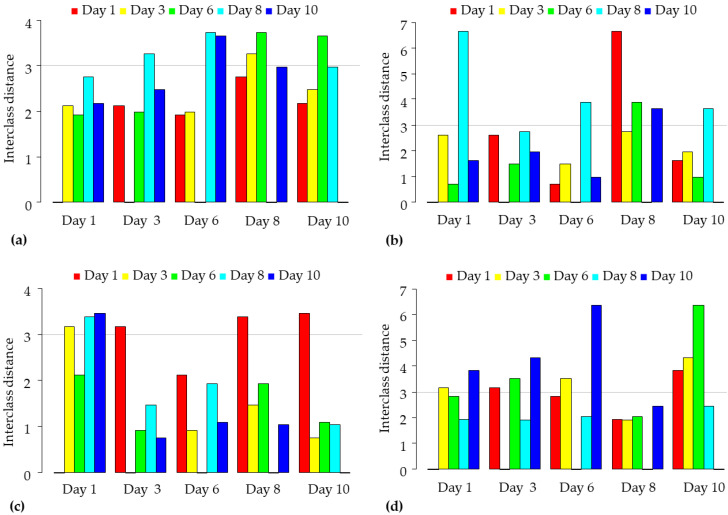
**The** SIMCA interclass distances according to storage time: (**a**) results of control ‘Kántorjánosi’ samples (*n* = 88, k = 3); (**b**) results of control ‘Újfehértói fürtös’ samples (*n* = 84, k = 2); (**c**) results of MAP ‘Kántorjánosi’ samples (*n* = 81, k = 1); (**d**) results of MAP ‘Újfehértói fürtös’ samples (*n* = 83, k = 3).

Figure 9 reports the SIMCA interclass distances of the ‘Kántorjánosi’ and ‘Újfehértói fürtös’ samples, packed as control or MAP, calculated to discriminate according to different storage days. In general, all the four groups evaluated showed cases where interclass distances were greater than 3 (i.e., a significant difference). The apparent error rates during prediction were as follows for the four groups, respectively: 0.36 (Figure 9a); 0.38 (Figure 9b); 0.25 (Figure 9c); 0.15 (Figure 9d). 

In the case of the ‘Kántorjánosi’ variety, the results do not present a clear separation of specific days; however, the MAP shows some kind of linear tendency on days 6, 8 and 10 compared to day 1, and significant interclass distances for days 3, 8 and 10 (Figure 9c). In the case of the other variety, no clear trend was observed for differences by storage day compared to the results recorded on the first day. Modified atmosphere packaging resulted in a decreasing trend in interclass distances on days 3, 6 and 8, and a significant difference for days 3 and 10 (Figure 9d). 

**Figure 10:**
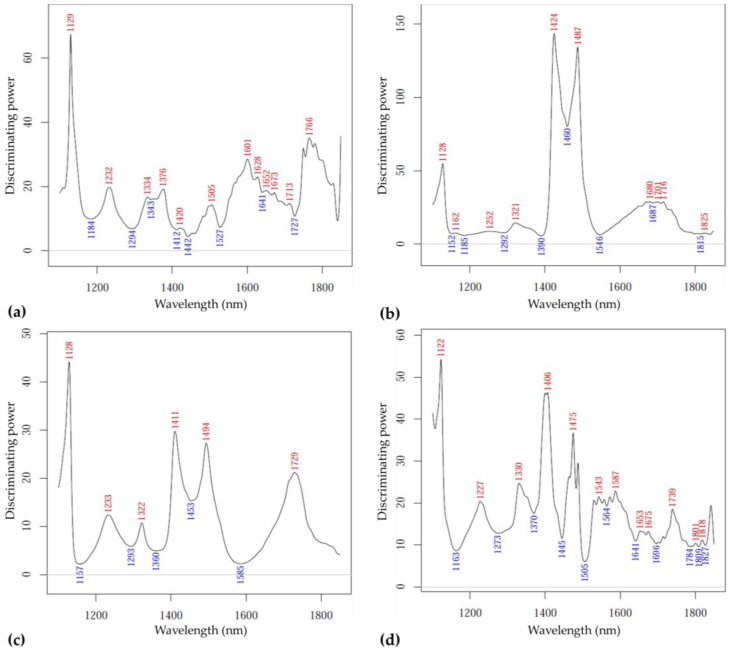
The SIMCA discriminating power plots for storage time: (**a**) control ‘Kántorjánosi’ samples (*n* = 88, k = 3); (**b**) control ‘Újfehértói fürtös’ samples (*n* = 84, k = 2); (**c**) MAP ‘Kántorjánosi’ samples (*n* = 81, k = 1); (**d**) MAP ‘Újfehértói fürtös’ samples (*n* = 83, k = 3).

Figure 10 presents the discriminating power plots corresponding to the above-detailed SIMCA modeling that allow the comparison of the influential variables. The bands at 1129, 1232, 1334, 1376, 1505, 1601–1713 and 1766 nm for the ‘Kántorjánosi’ samples packed as control (Figure 10a) and for the samples packed as MAP (Figure 10c) at 1228, 1232, 1322, 1411, 1494 and 1729 nm proved to be the most relevant. For the ‘Újfehértói fürtös’ samples packed as control, important bands were found at 1128, 1424, 1487, 1680–1716 nm (Figure 10b), while for the MAP samples these were at 1122, 1227, 1330, 1406, 1475, 1543–1587 and 1739 nm (Figure 10d). 

##### Discrimination according to Ripeness on the First and Last Days of Storage 

The soft independent modelling of class analogies was performed to classify spectral data according to the perceived ripeness level. For this, data were filtered to a specific variety and storage days independent of packaging and storage conditions to determine the extent to which samples differed between the initial and final day of storage. 

**Figure 11:**
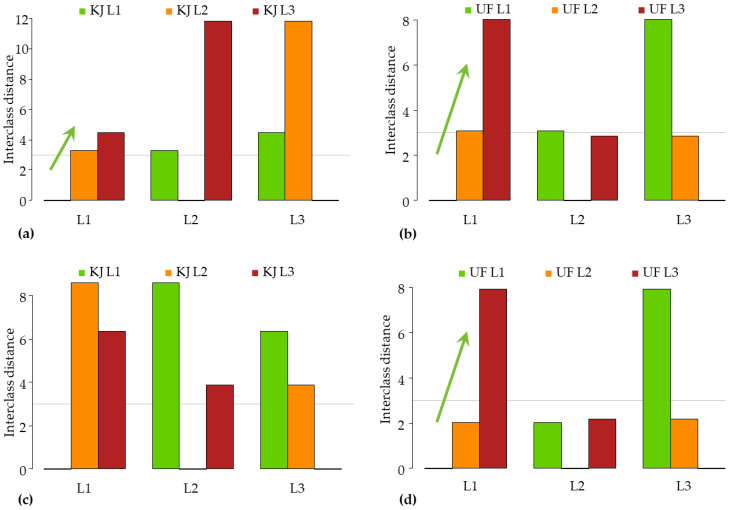
The SIMCA interclass distances according to ripeness: (**a**) results of ‘Kántorjánosi’ samples on day 1 (*n* = 28, k = 2); (**b**) results of ‘Újfehértói fürtös’ samples on day 1 (*n* = 33, k = 1); (**c**) results of ‘Kántorjánosi’ samples on day 10 (*n* = 33, k = 2); (**d**) results of ‘Újfehértói fürtös’ samples on day 10 (*n* = 33, k = 2).

Figure 11 reports the SIMCA interclass distances of the ‘Kántorjánosi’ and ‘Újfehértói fürtös’ samples of different ripeness on different storage days. It can be seen that all four sample sets showed significant differences among ripeness level, which was the smallest for the ‘Kántorjánosi’ samples on day 1. The apparent error rates during prediction were as follows for the four groups, respectively: 0.29 (Figure 11a); 0.13 (Figure 11b); 0.37 (Figure 11c); 0.13 (Figure 11d). 

The results showed a linear separation tendency based on ripeness and significant interclass distances between the samples analyzed on day 1 (Figure 11a,b). The interclass distances were greater for day 10, but with an opposite trend, and the apparent error rate was also higher here (Figure 11c). In the case of the ‘Újfehértói fürtös’ results on day 10, the interclass difference between L1 and L2 was shown to be not significant (Figure 11d). These outcomes can be attributed to the fact that the three ripeness levels were easier to develop from the initial sample set of the ‘Újfehértói fürtös’ variety, while this was not the case for ‘Kántorjánosi’. 

**Figure 12:**
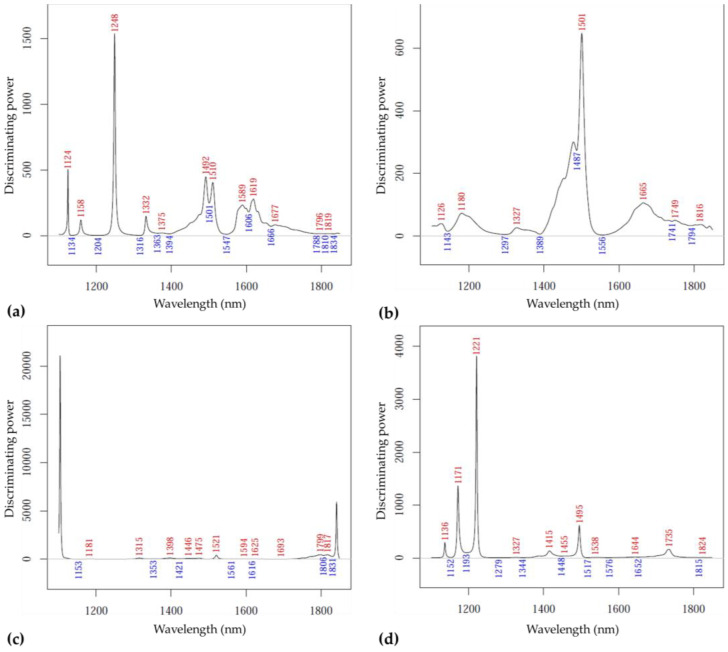
The SIMCA discriminating power plots for ripeness: (**a**) results of ‘Kántorjánosi’ samples on day 1 (*n* = 28, k = 2); (**b**) results of ‘Újfehértói fürtös’ samples on day 1 (*n* = 33, k = 1); (**c**) results of ‘Kántorjánosi’ samples on day 10 (*n* = 33, k = 2); (**d**) results of ‘Újfehértói fürtös’ samples on day 10 (*n* = 33, k = 2).

Figure 12 summarizes the power of the variables for discrimination according to perceived ripeness levels. For the data recorded on the ‘Kántorjánosi’ samples on day 1, bands at 1124, 1158, 1248, 1332, 1492, 1510, 1589, 1619 and 1677 nm (Figure 12a) were the most relevant, while for day 10 these were the bands around 1120, 1315, 1398, 1475, 1521, 1799–1817 nm. (Figure 12c). For the ‘Újfehértói fürtös’ variety on day 1, the following dominant wavelengths were found (Figure 12b): 1126, 1180, 1327, 1501, 1665, 1749, 1816 nm; for day 10: 1136, 1171, 1221, 1415, 1495 and 1735 nm (Figure 12d). 

##### Discrimination according to the Packaging on the First and Last Day of Storage 

The soft independent modelling of class analogies was performed to classify spectral data according to the mode of packaging (control or MAP). For this, data were filtered to a specific variety and storage days independent of ripeness level and storage condition to determine the extent to which samples differed between the initial and final day of storage as a result of different packaging. 

**Figure 13:**
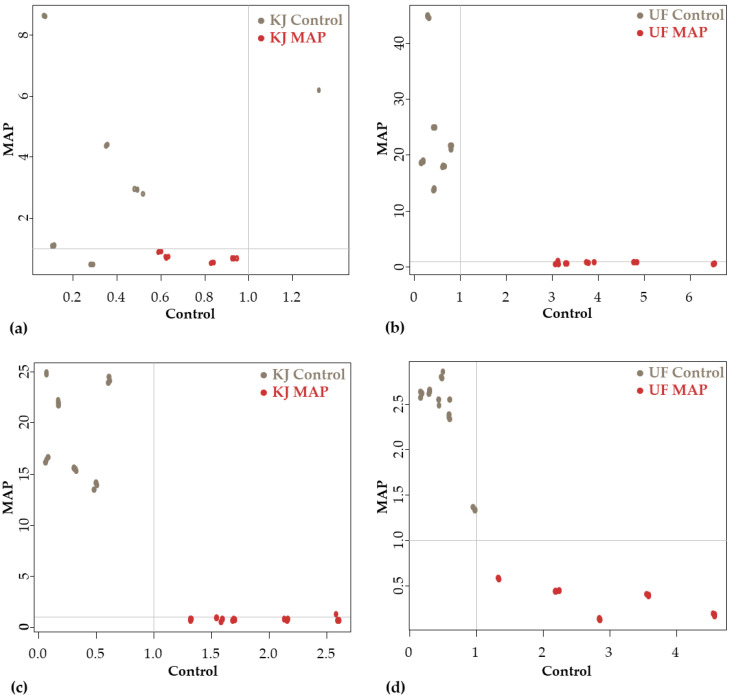
**The** SIMCA Cooman’s plots according to packaging: (**a**) results of ‘Kántorjánosi’ samples on day 1 (*n* = 28, k = 2); (**b**) results of ‘Újfehértói fürtös’ samples on day 1 (*n* = 33, k = 4); (**c**) results of ‘Kántorjánosi’ samples on day 10 (*n* = 33, k = 4); (**d**) results of ‘Újfehértói fürtös’ samples on day 10 (*n* = 33, k = 3).

Figure 13 shows the SIMCA results on Cooman’s plots of the ‘Kántorjánosi’ and ‘Újfehértói fürtös’ samples of different packaging on different storage days. The results demonstrated complete separation based on the spectra. Significant interclass distances were found in all cases, which were the following for the ‘Kántorjánosi’ and ‘Újfehértói fürtös’ varieties on day 1 and day 10, respectively: 4.29 (Figure 13a); 13.20 (Figure 13b); 6.65 (Figure 13c); 4.74 (Figure 13d). The apparent error rates during prediction were the following according to the previous order: 0.43; 0.56; 0; 0.13. The results confirm that the mode of packaging does have an influence on the behavior of the sample during storage. 

**Figure 14:**
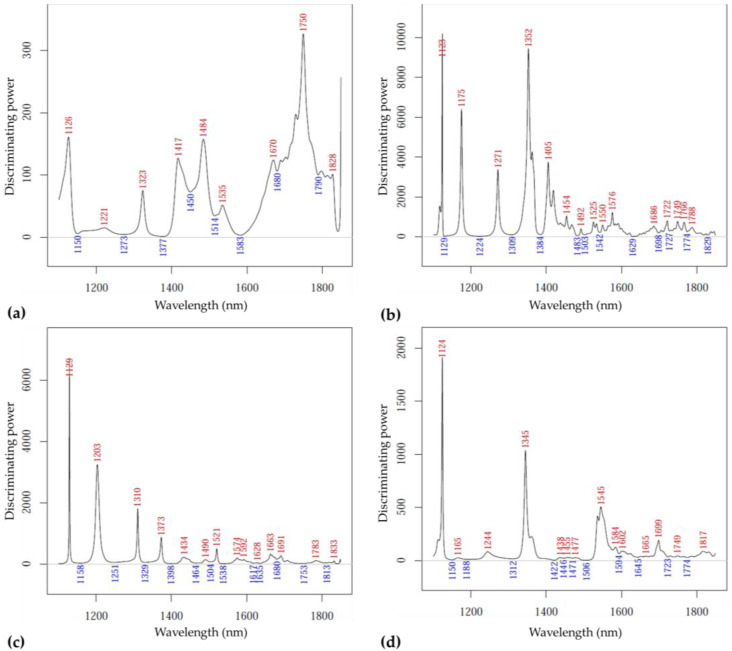
**The** SIMCA discriminating power plots for packaging: (**a**) results of ‘Kántorjánosi’ samples on day 1 (*n* = 28, k = 2); (**b**) results of ‘Újfehértói fürtös’ samples on day 1 (*n* = 33, k = 4); (**c**) results of ‘Kántorjánosi’ samples on day 10 (*n* = 33, k = 4); (**d**) results of ‘Újfehértói fürtös’ samples on day 10 (*n* = 33, k = 3).

Figure 14 summarizes the most significant contributing variables for the discrimination according to the mode of packaging: for the data recorded on ‘Kántorjánosi’ samples on day 1, bands at 1126, 1221, 1323, 1417, 1484, 1535, 1670, 1750 and 1828 nm (Figure 14a), and on day 10, bands at 1129, 1203, 1310, 1373, 1434, 1490, 1521, 1574, 1663 and 1691 nm proved to be the most relevant (Figure 14c). For the ‘Újfehértói fürtös’ variety on day 1, the following dominant wavelengths were found: 1123, 1175, 1271, 1352, 1405, 1454, 1492–1576, 1686, 1722–1788nm (Figure 14b); for day 10: 1124, 1244, 1345, 1545, 1584, 1699 and 1817 nm (Figure 14d). 

##### Discrimination according to Storage Condition on the First and Last Days of Storage 

The soft independent modelling of class analogies was performed to classify spectral data according to storage conditions (3 °C or 5 °C). For this, data were filtered to a specific variety and storage days independent of ripeness level and packaging to determine the extent to which samples differed between the initial and final days of storage as a result of different storage temperatures. 

Figure S5 shows the SIMCA results on Cooman’s plots of the ‘Kántorjánosi’ and ‘Újfehértói fürtös’ samples of different storage conditions on different storage days. Similar to the previous evaluations, the results demonstrated good separation based on the spectra. In all the cases, except for the data recorded on the ‘Kántorjánosi’ samples on day 1 (Figure S5a), significant interclass distances were typical. Presumably, this species needed more time to adapt to refrigeration. In the other sample sets, the interclass distances and apparent error rates during prediction were, respectively, as follows: 3.33 and 0.13 (Figure S5b); 8.7 and 0 (Figure S5c); 7.01 and 0 (Figure S5d). 

Figure S6 summarizes the most significant contributing variables for the discrimination according to storage temperature. For the data recorded on the ‘Kántorjánosi’ samples on day 1 the bands at 1128, 1237, 1323, 1388, 1452, 1474, 1534, 1591, 1726–1761 and 1801 nm (Figure S6a), and at day 10 the bands at 1125, 1195, 1273, 1369, 1460, 1491, 1537 and 1735 nm, proved to be the most relevant ones (Figure S6c). For the ‘Újfehértói fürtös’ variety on day 1, the following dominant wavelengths were found: 1127, 1211, 1322, 1353, 1494, 1526, 1591 and 1750 nm (Figure S6b); for day 10: 1125, 1201, 1305, 1370, 1463, 1547, 1609, 1691, 1720, 1786 and 1812 nm (Figure S6d). 

## 4. Conclusions 

The research presented here was built up from two successive, seemingly independent series of experiments. The first experiment compared washing and packaging methods to increase the shelf-life of two different varieties of sour cherries, based on the results obtained with reference methods. The treatment found to be appropriate based on the reference results was applied when the differences between samples during refrigerated storage were detected with NIR spectroscopy combined with SIMCA. 

Based on the results of the reference methods, it can be concluded that in the case of the ‘Kántorjánosi’ variety, two perforations should be used when modified atmosphere packaging is applied. Washing as a treatment demonstrated no significant difference. It is recommended to package the fruits in a modified atmosphere if weight loss during storage is to be avoided. It is also suggested to package the samples if one would like to significantly decrease the decay rate of the fruit. To avoid anaerobic fermentation, we chose two-perforation packaging for both types of cherries in Experiment II. As there was no significant difference between the measured parameters, only the microbiological results showed that washing reduces the total plate count in the short term. Since ozone treatment was not found to be effective in terms of maturation inhibition during storage, the samples in Experiment II were pretreated with tap water. 

Interestingly, it was found that the treatment and packaging did not result in significant differences in the change of Brix° during the storage of the ‘Kántorjánosi’ sour cherry. Regarding total plate count, it can be concluded that the quality of the washing treatment does influence the increase in surface TPC in the samples tested. 

The NIR spectral evaluations highlight that the fingerprinting approach generally distinguished the samples with good accuracy; however, the detectable differences were variety- and storage condition-dependent, and in many cases increased as storage progressed. The research findings suggest the further building of the initial database due to the experienced high variability of fruits as well as the correlation of NIR spectroscopy and reference parameters to support postharvest handling and the fast quality control of sour cherries. 

Hand-held NIR spectrometers are relatively cheaper than benchtop instruments, allow on-site inspections and are already available on the market. However, they are still mainly used in scientific and/or laboratory environments. With the extensive development of miniaturized devices and customer-friendly, cloud-based software, these devices could become widespread soon in commercial environments, and even among conscious consumers. With these investigations, our long-term goal is to develop an effective methodology to determine the ripeness of fruit for storage and its consumability, reducing the insecurity associated with perishable fruit. 

## Figures and Tables

**Table 1:** Sample treatment and packaging groups in Experiment I.

Variety	Treatment	Perforation Number	Marking
‘Kántorjánosi’ (KJ)	Control (K)	Without perf. (WOP)	KJ–K
‘Kántorjánosi’ (KJ)	Tap Water (W)	One piece of perf. (1P)	KJW-1P
‘Kántorjánosi’ (KJ)	Tap Water (W)	Two pieces of perf. (2P)	KJW-2P
‘Kántorjánosi’ (KJ)	Ozone Microbubble (O)	One piece of perf. (1P)	KJO-1P
‘Kántorjánosi’ (KJ)	Ozone Microbubble (O)	Two pieces of perf. (2P)	KJO-2P
‘Újfehértói fürtös’ (UF)	Control (K)	Without perf. (WOP)	UF–K
‘Újfehértói fürtös’ (UF)	Tap Water (W)	One piece of perf. (1P)	UFW-1P
‘Újfehértói fürtös’ (UF)	Tap Water (W)	Two pieces of perf. (2P)	UFW-2P
‘Újfehértói fürtös’ (UF)	Ozone Microbubble (O)	One piece of perf. (1P)	UFO-1P
‘Újfehértói fürtös’ (UF)	Ozone Microbubble (O)	Two pieces of perf. (2P)	UFO-2P

**Table 2:** Sample groups used in Experiment II.

Variety	Packaging	Storage Temperature	Ripeness
‘Kántorjánosi’	Control	~3 °C	L1
			L2
			L3
		~5 °C	L1
			L2
			L3
	MAP	~3 °C	L1
			L2
			L3
		~5 °C	L1
			L2
			L3
‘Újfehértói fürtös’	Control	~3 °C	L1
			L2
			L3
		~5 °C	L1
			L2
			L3
	MAP	~3 °C	L1
			L2
			L3
		~5 °C	L1
			L2
			L3

## Data Availability

The data presented in this study are available on request from the corresponding author. The data are not publicly available due to privacy concerns.

## Supplementary Materials

The following supporting information can be downloaded at: https://www.mdpi.com/article/10.3390/s23010479/s1, Figure S1. Decay rate during the storage of control and MAP: (a) ‘Kántorjánosi’ and (b) ‘Újfehértói fürtös’ sour cherries with different washing pretreatments; Figure S2. Change in L* color parameter during the control and MAP storage of ‘Újfehértói fürtös’ sour cherries with different washing pretreatments; Figure S3. Change in b* color parameter during the control and MAP storage of ‘Újfehértói fürtös’ sour cherries with different washing pretreatments; Figure S4. Raw spectra collected on ‘Újfehértói fürtös’ samples during the 10 days of storage: (a) Control samples stored at 3 °C; (b) Control samples stored at 5 °C; (c) MAP samples stored at 3 °C; (d) MAP samples stored at 5 °C. Average difference spectra of ‘Újfehértói fürtös’ samples calculated on storage days: (e) Control samples stored at 3 °C; (f) Control samples stored at 5 °C; (g) MAP samples stored at 3 °C; (h) MAP samples stored at 5 °C; Figure S5. The SIMCA Cooman’s plots according to storage temperature: (a) results of ‘Kántorjánosi’ samples on day 1 (*n* = 28, k = 10); (b) results of ‘Újfehértói fürtös’ samples on day 1 (*n* = 33, k = 3); (c) results of ‘Kántorjánosi’ samples on day 10 (*n* = 33, k = 4); (d) results of ‘Újfehértói fürtös’ samples on day 10 (*n* = 33, k = 4); Figure S6. The SIMCA discriminating power plots for storage temperature: (a) results of ‘Kántorjánosi’ samples on day 1 (*n* = 28, k = 10); (b) results of ‘Újfehértói fürtös’ samples on day 1 (*n* = 33, k = 3); (c) results of ‘Kántorjánosi’ samples on day 10 (*n* = 33, k = 4); (d) results of ‘Újfehértói fürtös’ samples on day 10 (*n* = 33, k = 4).
