# Supplementary material for: Application of Near Infrared Spectroscopy to Monitor the Quality Change of Sour Cherry Stored under Modified Atmosphere Conditions
Source: Sensors (Basel). 2023 Jan 2;23(1):479. doi: 10.3390/s23010479 (PMC9824794; doi:10.3390/s23010479)
Supplement: Supplementary file 1 [file sensors-23-00479-s001.zip › sensors-2087891-supplementary.pdf]

Article

Application of Near Infrared Spectroscopy to Monitor the Quality Change of Sour Cherry Stored under Modified Atmosphere Conditions

Gergo Szabo [1], Flora Vitalis [2], Zsuzsanna Horvath-Mezofi [1], Monika Gob [1], Juan Pablo Aguinaga Bosquez [2], Zoltan Gillay [2], Tamas Zsom [1], Lien Le Phuong Nguyen [3], Geza Hitka [1,*], Zoltan Kovacs [2] and Laszlo Friedrich [3]

[1] Department of Postharvest, Commerce, Supply Chain and Sensory Science, Institute of Food Science and Technology, Hungarian University of Agriculture and Life Sciences (MATE), H-1118 Budapest, Hungary
[2] Department of Food Measurement and Process Control, Institute of Food Science and Technology, Hungarian University of Agriculture and Life Sciences (MATE), H-1118 Budapest, Hungary
[3] Department of Livestock Product and Preservation Technology, Institute of Food Science and Technology, Hungarian University of Agriculture and Life Sciences (MATE), H-1118 Budapest, Hungary
* Correspondence: hitka.geza@uni-mate.hu

Supplementary Materials

3.1. Results of Experiment I.

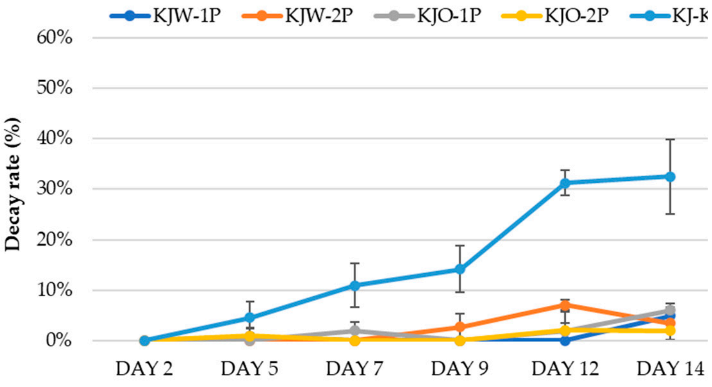

(a)

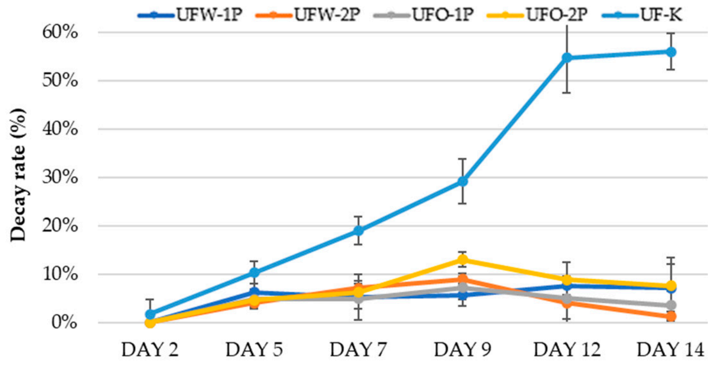

(b)

Figure S1. Decay rate during the storage of control and MAP **(a)** 'Kántorjánosi' and **(b)** 'Újfehértói fürtös' sour cherries with different washing pretreatments.

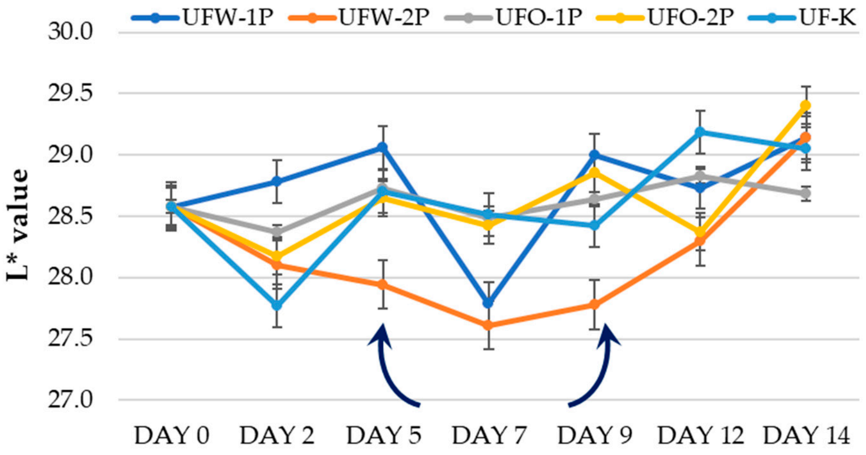

Figure S2. Change in L* color parameter during the control and MAP storage of 'Újfehértói fürtös' sour cherries with different washing pretreatments.

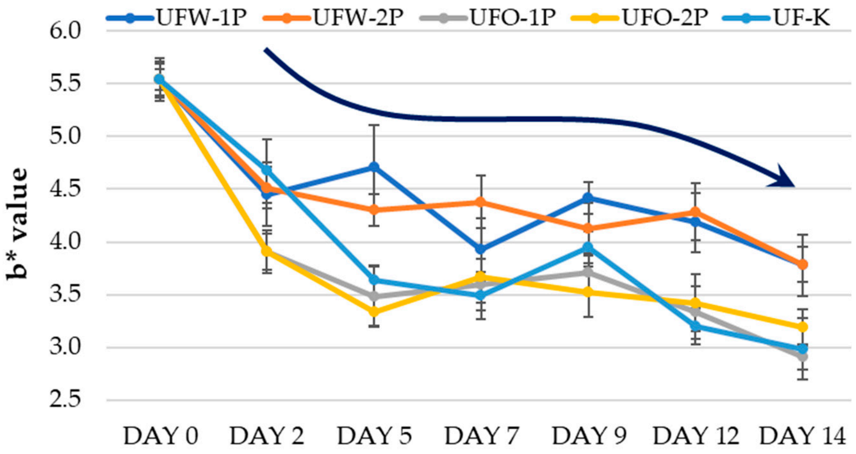

Figure S3. Change in b* color parameter during the control and MAP storage of 'Újfehértói fürtös' sour cherries with different washing pretreatments.

3.2. Results of Experiment II.

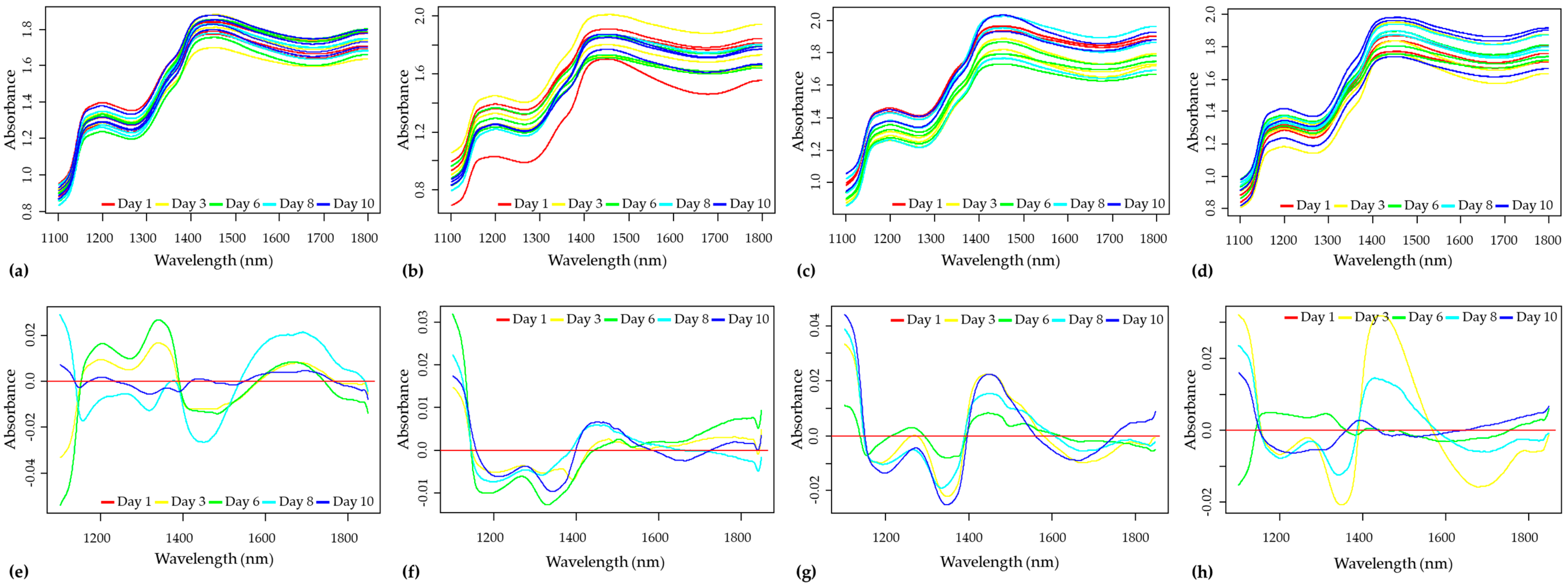

Figure S4. Raw spectra collected on ‘Újfehértói fürtös’ samples during the 10 days of storage: **(a)** Control samples stored at 3 °C; **(b)** Control samples stored at 5 °C; **(c)** MAP samples stored at 3 °C; **(d)** MAP samples stored at 5 °C. Average difference spectra of ‘Újfehértói fürtös’ samples calculated on storage days: **(e)** Control samples stored at 3 °C; **(f)** Control samples stored at 5 °C; **(g)** MAP samples stored at 3 °C; **(h)** MAP samples stored at 5 °C.

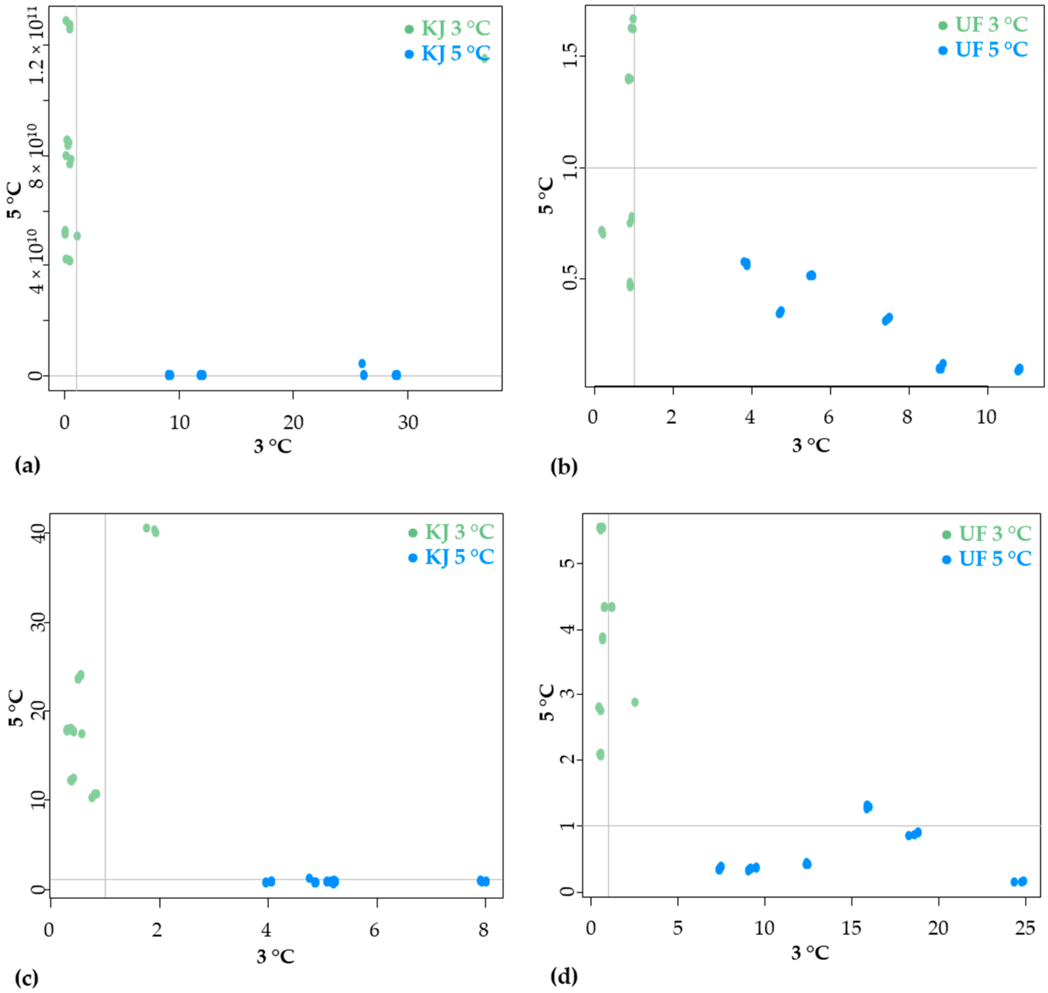

Figure S5. SIMCA Cooman's plots according to storage temperature: (**a**) results of 'Kántorjánosi' samples on day 1 ($n = 28$, $k = 10$) (**b**) results of 'Újfehértói fürtös' samples on day 1 ($n = 33$, $k = 3$); (**c**) results of 'Kántorjánosi' samples on day 10 ($n = 33$, $k = 4$); (**d**) results of 'Újfehértói fürtös' samples on day 10 ($n = 33$, $k = 4$).

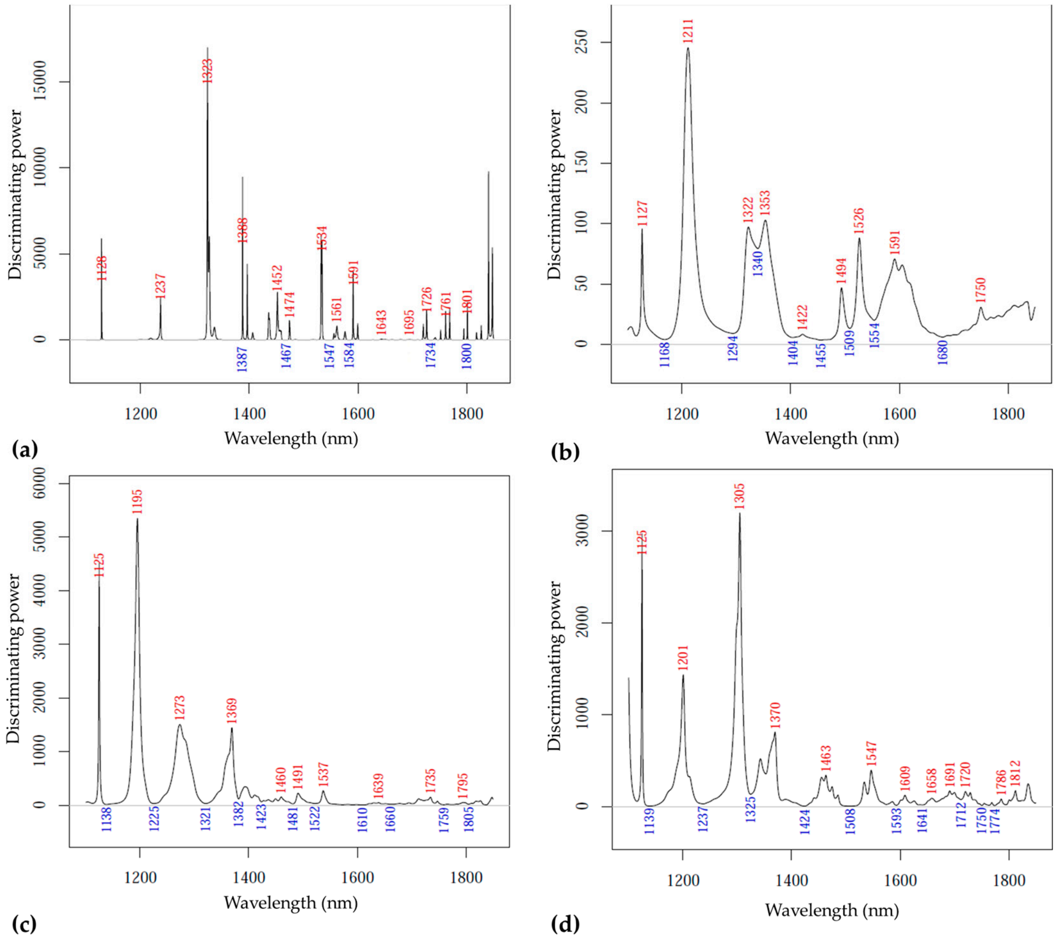

Figure S6. SIMCA discriminating power plots for storage temperature: (**a**) results of 'Kántorjánosi' samples on day 1 ($n = 28$, $k = 10$) (**b**) results of 'Újfehértói fürtös' samples on day 1 ($n = 33$, $k = 3$); (**c**) results of 'Kántorjánosi' samples on day 10 ($n = 33$, $k = 4$); (**d**) results of 'Újfehértói fürtös' samples on day 10 ($n = 33$, $k = 4$).
